# Psycholinguistic and emotion analysis of cryptocurrency discourse on X platform

**DOI:** 10.1038/s41598-024-58929-4

**Published:** 2024-04-13

**Authors:** Moein Shahiki Tash, Olga Kolesnikova, Zahra Ahani, Grigori Sidorov

**Affiliations:** Present Address: Instituto Politécnico Nacional (IPN), Centro de Investigación en Computación (CIC), Mexico, Mexico

**Keywords:** Cryptocurrency, Psycholinguistic, Digital coins, Reliability, Human behaviour, Computational science, Computer science, Scientific data

## Abstract

This paper provides an extensive examination of a sizable dataset of English tweets focusing on nine widely recognized cryptocurrencies, specifically Cardano, Binance, Bitcoin, Dogecoin, Ethereum, Fantom, Matic, Shiba, and Ripple. Our goal was to conduct a psycholinguistic and emotional analysis of social media content associated with these cryptocurrencies. Such analysis can enable researchers and experts dealing with cryptocurrencies to make more informed decisions. Our work involved comparing linguistic characteristics across the diverse digital coins, shedding light on the distinctive linguistic patterns emerging in each coin’s community. To achieve this, we utilized advanced text analysis techniques. Additionally, this work unveiled an understanding of the interplay between these digital assets. By examining which coin pairs are mentioned together most frequently in the dataset, we established co-mentions among different cryptocurrencies. To ensure the reliability of our findings, we initially gathered a total of 832,559 tweets from X. These tweets underwent a rigorous preprocessing stage, resulting in a refined dataset of 115,899 tweets that were used for our analysis. Overall, our research offers valuable perception into the linguistic nuances of various digital coins’ online communities and provides a deeper understanding of their interactions in the cryptocurrency space.

## Introduction

How people employ words daily can reveal a wealth of information about their beliefs, fears, thought processes, social connections, and personal characteristics^1^. Nowadays, online social media platforms significantly affect human life, and people freely pen their thoughts on social networks^2^. Furthermore, the extensive use of social media platforms has been instrumental in spreading awareness about groundbreaking projects. The proliferation of digital technologies has been facilitated by the process of globalization^3^. Owing to cryptocurrencies’ digital character, wide-ranging conversations occur in online forums and on social media platforms, including X (formerly known as Twitter) and Facebook. These platforms serve as significant determinants of the prevailing sentiment among the general public regarding cryptocurrencies and to a certain extent, influence their market valuations^4,5^. Notably, these networks are home to a huge user base, encompassing billions of individuals and a vast, intricate web of interconnected relationships among them^6^.

On January 29, 2021, Elon Musk, the world’s wealthiest individual at that time^7^, took a surprising step by adding the hashtag #bitcoin to his X bio. This unexpected move triggered an outpouring of excitement and prompted a surge in cryptocurrency enthusiasts rushing to buy Bitcoin. Remarkably, this seemingly minor action had a significant impact, quickly driving the price of Bitcoin from around $32,000 to over $38,000, ultimately leading to a remarkable increase of $111 billion in the cryptocurrency’s market capitalization^8^.

The majority of data produced on social networks is unstructured, making it challenging to quantify. As a result, it is typically analyzed using various characteristic features^9^. In the above instance, we observed a significant role that social media plays in shaping the cryptocurrency market.

Prior research in the field of cryptocurrency and blockchain technology has explored a wide range of subjects and methodologies. For instance, scholars have utilized natural language processing (NLP) techniques to analyze various aspects such as miner extractable value (MEV) in social media discussions^10^. Similarly, others developed strategies for maximizing wealth through Initial Coin Offerings (ICOs) in blockchain ventures^11^. Additionally, there have been endeavors to predict cryptocurrency prices and investigate the societal ramifications of these emerging technologies in contemporary business environments^12^. However, very few studies have addressed the psycholinguistics and emotions associated with the discourse on cryptocurrencies in social media. To contribute to these existing avenues of inquiry, our study aims to bridge a significant gap in the literature. Specifically, we intend to conduct psycholinguistic and emotion analyses, alongside with assessing the readability of cryptocurrency comments on social platforms, with NLP methods. By adopting this innovative approach, we seek to get more knowledge of the psychological and emotional dimensions in cryptocurrency discourse, which have thus far received limited attention in scholarly research. Our objective in this endeavor is twofold: to enhance awareness among newcomers in digital marketing to prevent misguided investments, and to offer support to traders who rely on metrics such as the fear and greed index in their trading strategies.

We analyzed nine distinct digital coins using psycholinguistic methods to assist cryptocurrency enthusiasts. The cryptocurrencies examined in this paper encompass Bitcoin^13^, Ethereum^14^, Ripple^15^, Binance^16^, Dogecoin^17^, Shiba^18^, Fantom^19^, Matic^20^, and Cardano^21^. Psycholinguistics is the examination of how linguistic elements and psychological aspects are interconnected. It is important to emphasize that we did not consider user-specific characteristics; our primary focus was solely on textual data. To clarify, we utilized psycholinguistic attributes that often convey the underlying meaning communicated by text. The text analysis we conducted comprises the following categories:

- 1. LIWC (Linguistic Inquiry and Word Count)^22^
- 2. Sentiment analysis^23^
- 3. Emotion analysis^24^
- 4. Assessment of readability^25–28^

Concerning the features we used for our computerized text analysis, first, we employed subcategories of LIWC^29^. We utilized only a selection of such subcategories, including Analytical Thinking, Clout, Drives, Affect, Money, Hope, Attention, Netspeak, and Filler. This internal dictionary encompasses an extensive compilation of more than 12,000 words, word stems, phrases, and specific emoticons. Each dictionary entry is associated with one or more categories, or subdictionaries, strategically designed to evaluate a wide range of psychosocial constructs^22^.

Investors typically initiate an assessment of public sentiment surrounding a particular cryptocurrency before making investment decisions^30^. Consequently, sentiment and emotion analysis in cryptocurrency markets has gained significant prominence^31^. Research indicates that tweets expressing positive sentiments can exert a substantial influence on cryptocurrency demand, and conversely, negative sentiments can have a similar effect^32,33^.

Readability refers to the level of ease with which a piece of writing can be understood or comprehended, primarily influenced by its writing style and presentation^34^. Readability not only relates to how easily a text can be understood with respect to its writing style but also takes into account how well readers comprehend it, read it at an appropriate speed, and find it engaging^35^.

Moreover, we went a step further by investigating the “reasons and significance” aspect. In simpler terms, we sought to determine which characteristics among the aforementioned four hold more importance for novice investors. To accomplish this, we explored the following research questions.

RQ1: Do psycholinguistic characteristics vary among digital coins?

RQ2: What are the dominant feelings expressed by X users regarding the cryptocurrencies under study?

RQ3: Does the readability level of tweets exhibit uniformity across all selected digital currencies?

RQ4: Is there any co-mention among different cryptocurrencies?

To address these research inquiries, we analyzed tweets related to nine distinct cryptocurrencies. We conducted psycholinguistic investigations and emotion analysis to respond to RQ1, RQ2, and RQ3 and extracted the above categories of features from the dataset, including LIWC, Readability, Sentiment, and Emotions analysis. To answer RQ4, we established a co-mention among different cryptocurrency coins, identifying which two coins tend to be mentioned together more frequently.

## Related work

A cryptocurrency is a form of digital currency designed for use as a means of exchange. It relies on robust cryptographic techniques to secure financial transactions, regulate the creation of additional units, and validate asset transfers^36^. Because of their substantial market values, cryptocurrencies have gained considerable interest, with some individuals regarding them as legitimate currencies and others as attractive investment prospects^33^.

### Sentiment and emotion analysis

Aslam et al.^37^ focused on sentiment analysis and emotion detection in cryptocurrency-related tweets collected using specific hashtags such as ’#cryptocurrency’, ’#cryptomarket’, and ’#Bitcoin’, amassing a total of 40,000 tweets. The authors employed traditional feature extraction methods like Bag-of-Words (BoW), TF-IDF, and Word2Vec, along with machine learning models including Random Forest (RF), Decision Tree (DT), k-Nearest Neighbors (KNN), Support Vector Machine (SVM), Gaussian Naive Bayes (GNB), and Logistic Regression (LR). Additionally, they leveraged advanced deep learning techniques, specifically a combination of Convolutional Neural Networks (CNN) and Long Short-Term Memory (LSTM) networks, to classify tweet sentiments as positive, negative, or neutral. Notably, they introduced an ensemble model that merges LSTM and Gated Recurrent Unit (GRU)^38,39^, achieving remarkable accuracy scores of 99% for sentiment analysis and 92% for emotion prediction.

Research in Ibrahim et al.^40^ centered on predicting early market movements of Bitcoin by harnessing sentiment analysis of X data^41,42^. The primary objective of their work was to introduce a Composite Ensemble Prediction Model (CEPM) built upon sentiment analysis. They employed a combination of data mining techniques, machine learning algorithms, and natural language processing to decipher public sentiment and mood states pertaining to cryptocurrencies. The research evaluated various models, such as Logistic Regression, Binary Classified Vector Prediction, Support Vector Machine, Naïve Bayes, and a single XGBoost^43^ for sentiment analysis. The remarkable point to be highlighted is the CEPM’s outperformance of other approaches, demonstrating its effectiveness in forecasting early Bitcoin market movements via the analysis of sentiment in X data.

Shahzad et al.^44^ presented a framework for performing sentiment analysis on X data with the aim of predicting the future price of Bitcoin. They highlighted the significance of NLP in bridging the gap between human communication and digital data and emphasized the growing importance of sentiment analysis in the field. The authors utilized three artificial intelligence tools, namely, LR, LSTM, and Deep Neural Network (DNN) Regressor to evaluate their performance in predicting Bitcoin prices. The best performance was demonstrated by the LSTM model.

Rahman et al.^45^ explored the usage of various natural language processing models for sentiment analysis in the context of cryptocurrency and financial market prediction. They used a dataset of approximately 100,000 news items, including tweets and Reddit posts, gathered from 77 public X timelines and Reddit subreddits over a six-month period from July to December 2021. The study also examined the creation of ensemble models, encompassing all 22 selected models as well as a subset of the top three models labeled as “ensemble (all) “ and “ensemble (top 3) “, which included Aigents+, Aigents, and FinBERT. The “ensemble (top 3)” method exhibited a higher degree of correlation with other models compared to the rest.

Huang et al.^46^ collected a substantial dataset comprising 24,000 cryptocurrency-related tweets and 70,000 comments from Sina-Weibo using specific keywords. The study adopted a methodology that utilized a training dataset consisting of posts from the top 100 crypto investor’s accounts on Sina-Weibo over the most recent seven days, while the subsequent day’s posts served as testing data. Remarkably, their sentiment analysis approach based on LSTM surpassed the time series auto-regression (AR) method by 87.0% in precision and 92.5% in recall.

The authors of^47^ aimed to detect sentiment and emotion in X posts and utilized this information for recommendations. They used a dataset containing tweets and user data and manually annotated 7,246 tweets and replies. Their approach involved text preprocessing and applying a Naïve Bayes classifier with cross-validation. The findings demonstrated that analyzing the entire text provided superior accuracy compared to focusing on specific words (NAVA). Moreover, as the number of cross-validation folds increased, the accuracy showed improvement. Specifically, in the realm of emotion analysis, the Naïve Bayes classifier achieved an accuracy of 47.34%. Furthermore, in sentiment analysis, Naïve Bayes outperformed other classifiers significantly, attaining an accuracy of 66.86%.

The researchers in^48^ utilized the AIT-2018 dataset^49^ to construct a model for detecting emotions expressed in tweets. The dataset of tweets was acquired through the X API by extracting tweets containing emotion-related hashtags such as ’#angry’, ’#annoyed’, ’#panic’, ’#happy’, ’#love’, and ’#surprised’. The proposed model integrated lexical-based approaches, employing emotion lexicons like WordNet-Affect and EmoSenticNet, along with supervised classifiers to autonomously classify multi-class emotions from the dataset. The authors conducted experiments employing three machine learning classifiers: Naïve Bayes, DT, and SVM. Their findings demonstrated that when filtering tweets using EmoSenticNet words, the precision in detecting emotions significantly improved. Specifically, the SVM classifier achieved a high precision rate of 89.28% in the Anger class, surpassing previous results obtained using logistic regression.

### Psycholinguistic analysis

Psycholinguistics utilizes various methods to comprehend language in the context of psychological processes. These methods encompass observational research, analysis, experimental studies, and the application of neuroimaging techniques^50^. Researchers also make use of text analysis models to interpret findings related to the language system. This section explores the methodologies employed by researchers in this field. Butt et al.^29^ presented a comprehensive analysis of the psycholinguistic aspects of rumors on online social media (OSM). Using the PHEME dataset^51^, which encompasses nine breaking news events, the researchers examined source tweets (rumor and non-rumor) and response tweets. They integrated various psycholinguistic features, including LIWC, SenticNet^52^, readability indices, and emotions to uncover user behavior patterns. Rumor source tweets were found to be characterized by language related to the past, prepositions, and motivations associated with reward, risk, and power. In contrast, non-rumor source tweets exhibited affective and cognitive processes, present-oriented language, and motivations linked to affiliation and achievement. Emotional analysis revealed that non-rumor tweets tended towards neutrality, while rumor-source tweets evoked fear and grief, subsequently prompting anger and fear in reactions.

Narman et al.^53^ reported an analysis of Reddit comments employing seven readability techniques to discern the education levels of users interested in eight cryptocurrencies. The data collection process involved gathering comments data from subreddits of eight cryptocurrencies by selecting ten to seventy top posts for each coin to collect distinct usernames. For education level information, they used Reddit.com to gather and categorize the collected comments data. The analysis was performed using seven text readability techniques. Interestingly, the results indicate that a majority, approximately 60%, possess an education level equivalent to middle school, with 30% at the high school level, while the remaining 10% span other educational levels.

The researches in^54^ aimed to assess the readability of tweets for English language learners. Their task involved collecting a dataset of 14,659 tweets and obtaining readability judgments from participants representing different language groups. For methodology, they analyzed various linguistic and content-related factors in the tweets, including emojis, hashtags, mentions, and links, as well as traditional readability measures like Flesch Reading Ease and Dale-Chall scores. The results revealed that demographic factors, such as language proficiency and education, were stronger predictors of tweet readability than any other single feature.

The proposal in^55^ included a framework to analyze linguistic features and cultural distinctions in climate-related tweets from the UK and Nigeria. A dataset of 81,507 English-language tweets was collected, comprising 44,071 from the UK and 37,436 from Nigeria. The study combined transformer networks with linguistic feature analysis, including the application of the (LIWC-22) software^56^,version 15.0, to address small dataset limitations and identify cultural differences^22^. Findings reveal that Nigerians tend to use more leadership language and informal words in climate change discussions on X, emphasizing the urgency of the issue. In contrast, UK discourse on climate change is characterized by more formality, logic, and longer words per sentence. The study also confirmed the geographical attribution of tweets using DistilBERT^55^, achieving an 83% accuracy rate.

## Dataset

This section provides a detailed overview of the data acquisition processes we employed. We clarify the exact steps undertaken during preprocessing and explore the complexities of conducting co-mention analyses among various cryptocurrency coins.

### Data collection

The data collection process commenced with the acquisition of X data pertaining to nine popular cryptocurrencies: Cardano, Bitcoin, Binance, Dogecoin, Ethereum, Fantom, Matic, Shiba, and Ripple^57^. These specific cryptocurrencies were selected for inclusion in the dataset due to their widespread usage across various research studies conducted by different scholars^18–20,58,59^. This endeavor yielded a substantial dataset comprising 832,559 tweets spanning from September 2021 to March 2023. After undergoing essential preprocessing steps, the dataset available for analysis was refined, resulting in a curated dataset consisting of 115,899 tweets. Table 1 presents dataset statistics both before and after preprocessing. Additionally, it lists the names of the coins and their respective symbols, which we utilized as keywords for extracting tweets from X. This extraction process was conducted separately for each coin, using both the name and the symbol as search criteria.

**Table 1:** Cryptocurrency names along with their symbols, their counts, before and after preprocessing.

Name of coin	Number of coins before preprocessing	Number of coins after preprocessing	Keywords1	Keywords2
Cardano	100,002	17,471	‘Cardano’	‘Ada’
Fantom	64,028	17,955	‘Fantom’	‘Ftm’
Matic	81,581	6806	‘Matic’	‘Matic’
Bitcoin	100,002	17,656	‘Bitcoin’	‘Btc’
Shiba	100,002	3445	‘Shiba’	‘Shib’
Dogecoin	90,538	5248	‘Dogecoin’	‘Doge’
Ripple	100,226	24,157	‘Ripple’	‘Xrp’
Ethereum	100,002	11,256	‘Ethereum’	‘Eth’
Binance	96,178	11,905	‘Binance’	‘Bnb’
Total	832,559	115,899		

### Data preprocessing

The utilization of the Tweepy^60^ API was instrumental in our tweet data collection procedure, as it empowered us to filter tweets according to diverse criteria, including date, location, language, and various tweet attributes, for example, the number of retweets. In the final phase, we focused exclusively on English-language tweets, excluding unnecessary fields such as ’username’, ’id’, ’date’, ’likeCount’, and ’retweetCount’ retaining only the actual tweet content. After obtaining the dataset, we conducted a multi-step data preprocessing procedure to refine and enhance the data. This procedure involved the following key steps:

URL Removal: We applied a regular expression pattern to identify and subsequently remove any URLs. Text Cleaning: This step included the removal of special characters, such as punctuation marks, with the assistance of a designated dictionary of special characters. Additionally, we excluded words that had a length less than or equal to two characters. The result was a cleaned version of the text data.

### Data labeling

In the process of data labeling, we examined each tweet systematically, with the primary objective of identifying any references to the selected cryptocurrencies. Notably, the search encompassed both “Bitcoin” and “Btc” in a case-insensitive manner, with any discovery leading to the classification of the tweet as Btc. This procedure was iteratively applied to all cryptocurrencies listed in Table 1, encompassing both their complete nomenclature and associated abbreviations.

Further, we encountered instances where tweets discussed multiple cryptocurrencies simultaneously which was uncovered as co-mention among these cryptocurrencies. The results of this co-occurrence analysis are considered in Section 3.4. To tackle this challenge effectively, a comprehensive set encompassing the names of all pertinent cryptocurrencies was devised. For instance, to annotate tweets as of Bitcoin, tweets mentioning any other cryptocurrency present in this predefined set were systematically excluded. The set itself comprised a roster of cryptocurrency names, notably including “Cardano”, “Ada”, “Fantom”, “Ftm”, “Matic”, “Shiba”, “Shib”, “Dogecoin”, “Doge”, “Ripple”,“Xrp”, “Ethereum”, “Eth”, “Binance” and, “Bnb“.

Subsequently, the inclusion of both Bitcoin and Btc into this enumerated list facilitated the resolution of similar issues encountered with other cryptocurrencies, with the same process being replicated across each cryptocurrency to ensure comprehensive data labeling. As an example, in the dataset, a tweet was identified as featuring the keyword Matic. The content of the tweet is provided below: APompliano Good day sir, I have 100$ to invest in a coin right, small but what I can afford for now. So, Im thinking It I should rather go for $ada $matic, $doge What do you suggest fam.

This tweet was acquired using the keyword Matic, and the keywords to be examined for the Matic coin included:’Cardano’, ’Ada’, ’Fantom’, ’Ftm’, ’Bitcoin’, ’Btc’, ’Shiba’, ’Shib’, ’Dogecoin’, ’Doge’, ’Ripple’, ’Xrp’, ’Ethereum’, ’Eth’, ’Binance’ and, ’Bnb’. The exclusion criterion described above ensured that if any of these keywords were present in a tweet, except for the keywords related to the specific coin for which we used keywords to extract tweets, that tweet should be removed. In our example, it’s evident that the tweet contains both ’ada’ and ’doge’ keywords, indicating that it should be removed.

**Figure 1:**
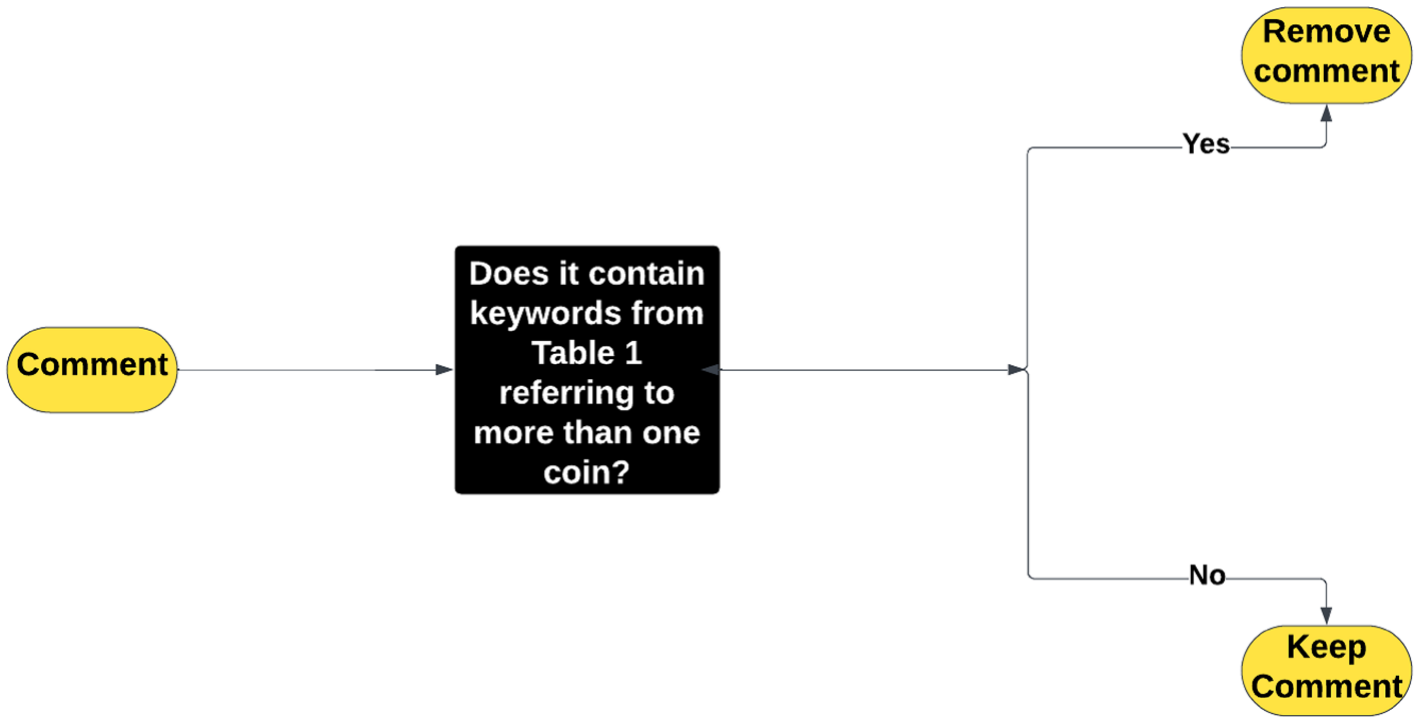
Example tweet processing.

Figure 1 illustrates the processing steps for a tweet.

### Cryptocurrencies co-mention

During the labeling process, we examined co-mention and co-occurrence among various cryptocurrencies in tweets. Such analysis resulted in an interesting observation: multiple cryptocurrencies often co-occurred in the same tweets, indicating a significant level of co-mention, which led us to reconsider our labeling model, as previously detailed.

In this section, we explore specific, noteworthy co-mentions among cryptocurrency coins. These co-mentions provide valuable information for our investigation, enhancing our understanding of the relationships and trends emerging in the cryptocurrency ecosystem as reflected in social media discourse. The co-mention matrix provided in Table 2 serves as a tool for assessing the relationships between different cryptocurrencies, particularly concerning their trends and market dynamics, as opposed to a sole focus on price movements. An illustrative example lies in the substantial positive co-mention of Bitcoin (Btc) and Ethereum (Eth) in 53.52% of tweets. This significant co-occurrence indicates that when Bitcoin undergoes an upward trend, or garners increased market attention, Ethereum frequently follows suit. This co-occurrence can be attributed to the prominent positions both cryptocurrencies occupy in the market, as well as to their substantial influence on overall market sentiment.

**Table 2:** Co-mention matrix of cryptocurrencies (%).

	Ethereum	Binance	Bitcoin	Shiba	Ripple	Matic	Fantom	Cardano	Dogecoin
Ethereum		37.98	53.52	0.54	38.80	23.08	5.80	28.22	20.93
Binance	37.98		37.00	0.39	33.55	23.07	4.46	23.16	17.72
Bitcoin	53.52	37.00		0.39	35.02	18.60	5.06	27.10	20.44
Shiba	0.54	0.39	0.39		0.30	0.37	0.19	0.31	0.44
Ripple	38.80	33.55	35.02	0.30		17.50	2.78	23.85	17.37
Matic	23.08	23.07	18.60	0.37	17.50		4.71	11.33	13.56
Fantom	5.80	4.46	5.06	0.19	2.78	4.71		3.21	2.88
Cardano	28.22	23.16	27.10	0.31	23.85	11.33	3.21		12.72
Dogecoin	20.93	17.72	20.44	0.44	17.37	13.56	2.88	12.72	

In contrast, co-mention values nearing 0% in Table 2 signify a lack of substantial co-occurrence among cryptocurrencies. This absence of mention underscores the potential for diversification strategies for designing a cryptocurrency portfolio. Cryptocurrencies exhibiting low or negative co-mention can be strategically employed to diversify a portfolio, potentially resulting in reduced overall portfolio risk. Conversely, cryptocurrencies demonstrating high positive co-mention may offer limited diversification benefits as they tend to move in sync with one another.

Inside the domain of cryptocurrency portfolio management and risk mitigation, these co-mention observations underscore the critical importance of accurate asset selection and allocation, especially in light of the observed co-mention among cryptocurrencies. Such strategic decision-making becomes paramount in achieving diversified and risk-optimized cryptocurrency portfolios.

## Analyses

In this research, we examined cryptocurrency data, concentrating on a specific group of cryptocurrencies. Our choice of these particular coins was driven by their significant popularity among users, as well as the limited availability of substantial data for other coins. To interpret the data, we applied four analytical methods explained in section "Introduction". Here we present the outcomes of our analysis for each of the aforementioned cryptocurrencies. The selection of features was made considering their past influence^29,61^. In the analysis conducted, LIWC assessments were applied to nine cryptocurrencies, resulting in an extensive collection of nine distinct analyses. We selected values that were highly informative for extracting linguistic interpretations relevant to cryptocurrencies. Our choice was made to capture key aspects of sentiment, linguistic style, and thematic content pertinent to discussions around cryptocurrencies. By narrowing down our focus to these particular features, we aimed to mine information from the psychological and linguistic dimensions of cryptocurrency discourse, thus aligning analysis with our goals. these categories encompass analytical thinking (metric of logical, formal thinking), clout (language of leadership), drives (related to personal motivations and psychological desires), affect (linguistic expressions associated with emotional and affective states expressed by a given text), money (refers to a set of linguistic cues or indicators related to financial terms, wealth, and economic aspects, Want (a human ability that allows individuals to envision future events with flexibility), attention (crucial subset of the “Perception” category), netspeak (represents a subset of the conversational category) and filler (non-essential sounds, words, or phrases, commonly used in speech to fill in pauses and maintain the flow of conversation without altering its meaning). In the drives and affect categories, additional features will be elaborated upon in the following discussion. Our examination indicated that Fantom attracts a larger number of tweets centered on technical aspects and holds a higher level of trust in comparison to other cryptocurrencies. For Binance, our observations revealed that the tweets predominantly revolve around themes of affiliation, achievements, and the pursuit of power and wealth. This pattern in discussions on Binance suggests a focus on notable accomplishments and financial success, indicative of a unique narrative and sentiment surrounding the coin. For Matic, the tweets primarily center around emotional impact compared to other cryptocurrencies. This emphasis on affective responses suggests that the coin is particularly influenced by emotional novelty. This distinctive characteristic could be considered a contributing factor to the fluctuations in the coin’s price, as emotional sentiment plays a significant role in shaping market dynamics and investor behavior. Our analysis revealed that Dogecoin exhibits a higher prevalence of netspeak, the informal language commonly used on the internet, compared to other cryptocurrencies. Conversely, Ethereum appears to attract more attention relative to other coins. This distinction suggests that Dogecoin is characterized by a more casual and internet-centric communication style, while Ethereum stands out for its ability to capture increased Attention and interest. A deeper understanding of the communication dynamics and community sentiment surrounding different coins may aid investors in making more informed choices, aligning their investment strategies with the unique qualities and trends associated with each cryptocurrency. From an emotional perspective, most cryptocurrencies exhibit a generally moderate and harmonious emotional profile. Notably, there is a distinct focus on the emotional category of Anticipation, with Dogecoin taking the forefront in this aspect. In this context, Anticipation likely signifies the expectation or excitement surrounding the future prospects, developments, or events associated with these cryptocurrencies.The outcomes of our analysis are presented in Table 5. In terms of readability, the analysis revealed that Dogecoin’s tweets are relatively more challenging to read and comprehend, as indicated by lower scores on the Flesch Reading Ease measure. The Flesch-Kincaid and Dale-Chall Measures suggest an average reading difficulty level akin to content tailored for college graduates. Conversely, Ethereum’s tweets, as per the Gunning Fog Index, demand a higher level of reading proficiency, indicating a more complex and advanced readability suitable for individuals with a college-level education and vocabulary. To explore additional results, refer to Figs. 5 and 6s, as well as Table 6.

### LIWC

The LIWC model revolutionized psychological research by making the analysis of language data more robust, accessible, and scientifically rigorous than ever before. LIWC-22 examines over 100 textual dimensions, all of which have undergone validation by esteemed research institutions globally. With over 20,000 scientific publications utilizing LIWC, it has become a widely recognized and trusted tool in the field^62^ giving way to novel approaches in analysis^63,64^. Although LIWC provides several benefits, it has its limitations. One drawback is its dependence on predefined linguistic categories, which might not encompass nuances and variations present in natural language. Furthermore, LIWC may encounter challenges in accurately deciphering sarcasm, irony, and other subtle forms of language usage, potentially resulting in text misinterpretation.

To effectively convey the outcomes of our analysis, average values among all the tweets were computed for each of LIWC categories. Averages can help identify broadscale sentiment trends over time. By tracking changes in average scores across key linguistic categories, such as sentiment, emotion, or cognitive processes, one can observe shifts in user sentiment and attitudes towards cryptocurrencies, market developments, or external events. Therefore, the average was calculated by summing up the scores of all comments related to each coin for each LIWC feature and then dividing by the total number of comments for that coin. These computed averages provide information along the linguistic and psychological dimensions intertwined with the selected digital currencies. A comprehensive presentation of these average values for each category can be found in Table 3.

#### Analytical thinking and clout

Analytical Thinking, when showing high numerical values, signifies a formal, logical, and hierarchical thought process. Conversely, lower numbers suggest a more informal, personal, present-focused, and narrative style of thinking^65^. The values of this category computed for tweets related to cryptocurrency, reach their highest average score of 67.76 in texts mentioning Fantom. This fact indicates that, on average, discussions in this domain exhibit a relatively high level of logical and formal thinking. Conversely, the lowest average score of 52.00 was found for Ripple, which might suggest that discussions concerning this particular cryptocurrency place slightly less emphasis on logical and analytical thinking compared to the cryptocurrency domain’s average.

**Figure 2:**
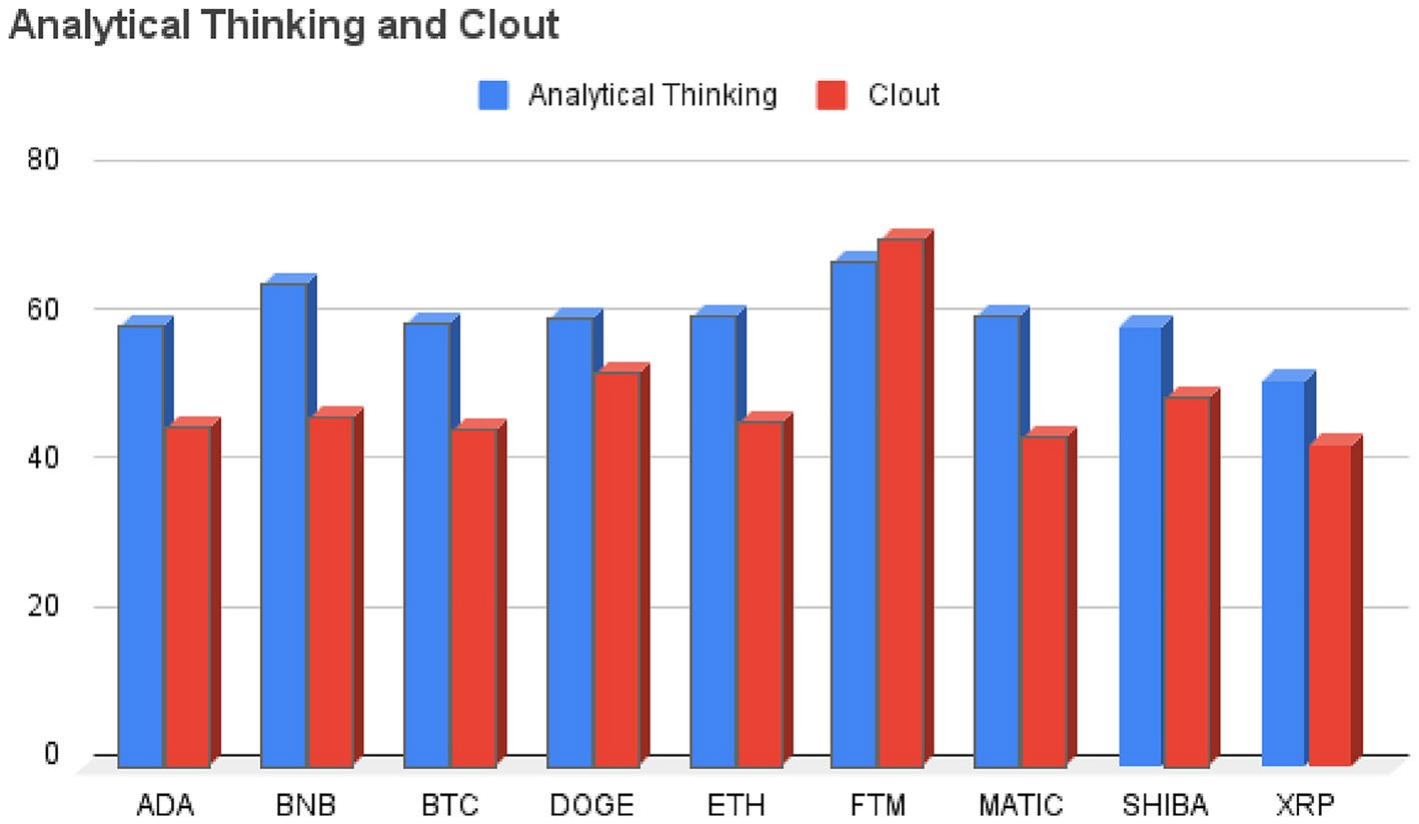
Comparative evaluation of analytical thinking and clout scores across different cryptocurrencies.

Clout is one of the four summary variables in LIWC designed to assess the degree of confidence and certainty conveyed in the text^66,67^. Our analysis revealed that the cryptocurrency Fantom exhibits a relatively high Clout score, with an average result of 70.91. This suggests that discussions and conversations related to Fantom often convey a strong sense of confidence and certainty. This high Clout score may also indicate a substantial degree of assurance in Fantom stability. In contrast, the cryptocurrency Ripple demonstrates a comparatively lower Clout score with an average result of 43.39. Figure 2 presents a comparative evaluation of Analytical Thinking and Clout scores across different cryptocurrencies. This suggests that discussions related to Ripple may not consistently display the same level of confidence and certainty found in the Fantom discussions. In essence, when Fantom demonstrates higher Clout values, it signifies that the users who composed the tweets are expressing increased confidence. This, in turn, leads us to infer a heightened level of knowledge on their part. In both analyses, we observed that Fantom consistently had the highest scores, indicating a higher level of analytical thinking and confidence in discussions related to it. Conversely, Ripple consistently had the lowest scores in both categories, suggesting a relatively lower emphasis on analytical thinking and a lower degree of expressed confidence in discussions related to it. While these observations suggest a correlation between analytical thinking and confidence in these specific cryptocurrency discussions, it’s important to note that correlation does not imply causation. Other factors, such as market conditions, community sentiment, and news events, can also influence these results. For example, when we examined Binance, we foound that it ranks as the second-highest in terms of Analytical Thinking scores among the various cryptocurrencies. However, when we assess it as the position in the Clout category, Binance ranks fifth. The results of Analytical Thinking and Clout analysis related to digital currencies can be viewed in Table 3.

**Table 3:** LIWC Analysis of Digital Coins.

Liwc analysis\digital coins	Cardano	Binance	Bitcoin	Dogecoin	Ethereum	Fantom	Matic	Shiba	Ripple
Analytical Thinking	59.27	64.97	59.64	60.23	60.38	67.76	60.65	59.25	52.00
Clout	45.55	46.85	45.37	52.79	46.33	70.91	44.43	49.46	43.39
Drives	2.94	3.16	3.15	2.59	3.14	2.38	3.08	2.93	2.86
Affect	3.93	4.02	4.62	4.18	4.00	3.99	5.20	4.60	4.12
Money	9.33	10.51	6.69	4.53	8.45	8.54	9.32	2.46	8.52
Want	0.20	0.20	0.25	0.26	0.35	0.19	0.28	0.41	0.27
Attention	0.60	0.62	0.59	0.25	0.72	0.53	0.40	0.49	0.43
Netspeak	0.82	0.56	0.82	1.06	0.80	7.76	0.83	0.91	0.88
Filler	0.03	0.02	0.04	0.10	0.03	0.02	0.04	0.10	0.03

#### Drives and affect

**Figure 3:**
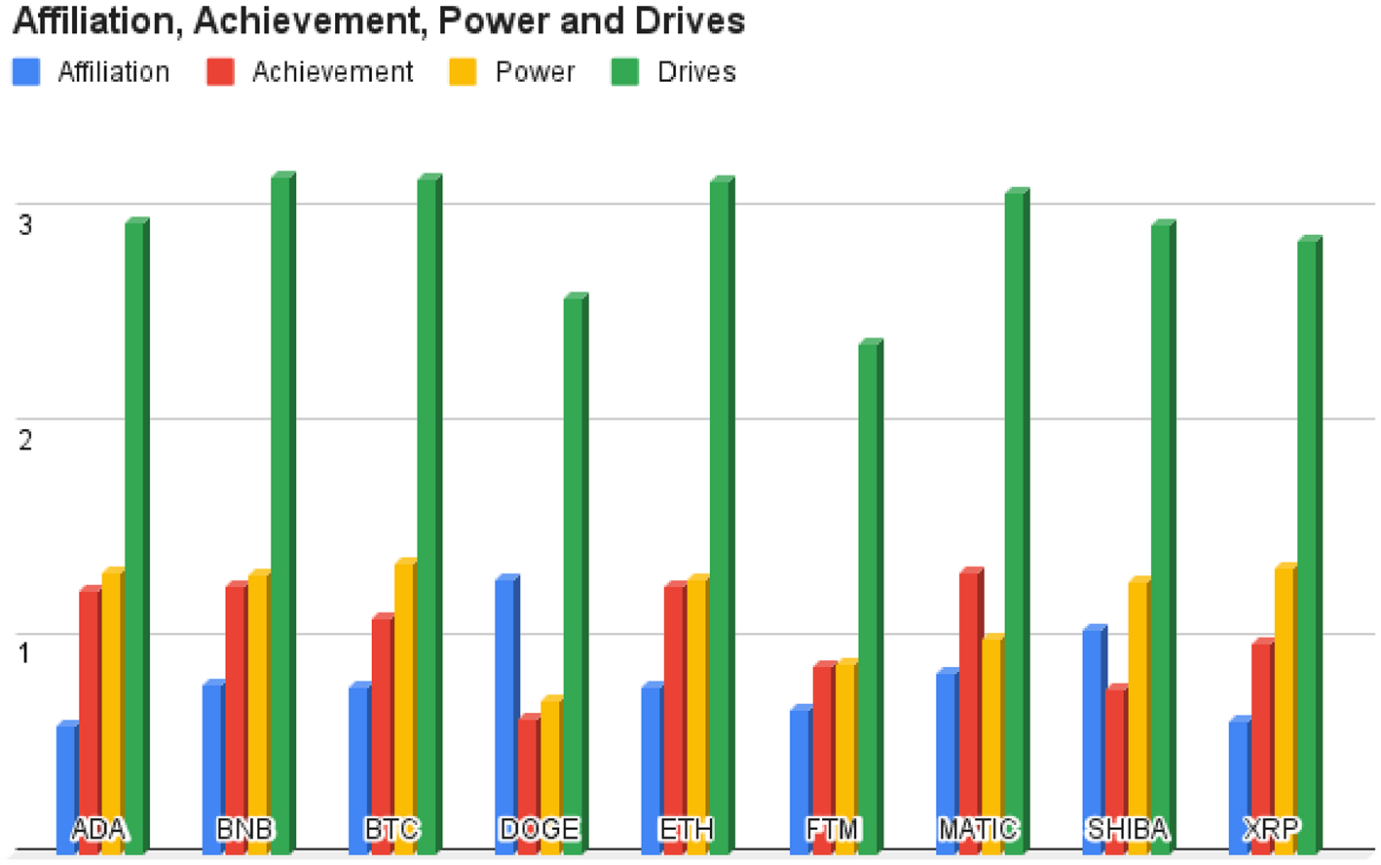
Frequency of language associated with affiliation, achievement, power, and drives across different cryptocurrency discussions.

Drives is a comprehensive dimension that encapsulates various needs and motives^65^. In our LIWC analysis, we concentrated on the Drives, particularly examining the aspects of Affiliation, Achievement, and Power. We observed that the presence of Affiliation-related language (such as “us” and “help“) is comparatively lower in discussions related to Cardano, while it appears more frequently in conversations about Dogecoin. Similarly, in terms of Achievement-related language (including “work”, “better”, and “best“), Dogecoin tends to have fewer instances compared to Matic. Furthermore, when examining Power-related language (like “allow” and “power“), we found that Dogecoin exhibits a lower frequency, while Bitcoin discussions tend to feature a greater occurrence of such language. These patterns highlight variations in linguistic expressions across different cryptocurrencies, shedding light on the distinctive characteristics of discussions over different digital coins. Upon closer examination, it became evident that tweets originating from Binance sources tended to include a higher frequency of words associated with Drives, whereas Fantom source tweets had a notably lower occurrence of Drives-related words. Additional details can be found in Fig. 3.

**Figure 4:**
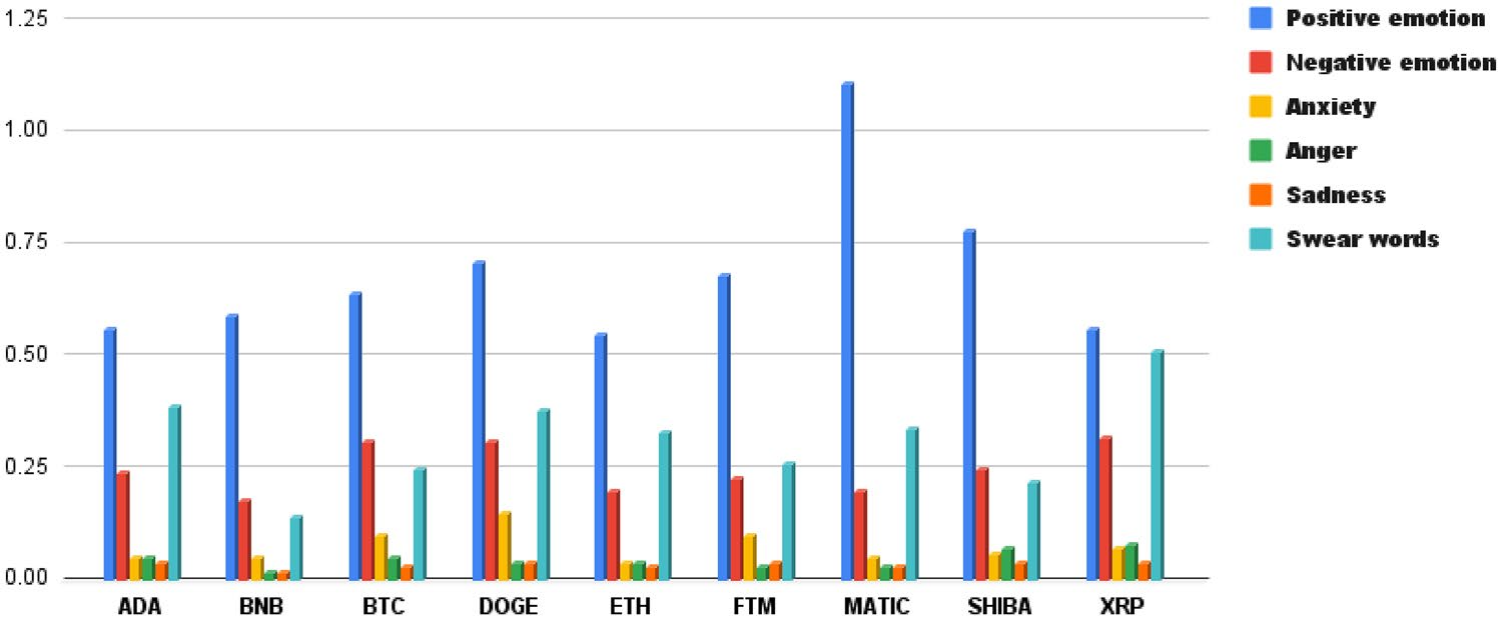
Comparative analysis of affective language dimensions-positive tone, negative tone, emotion, and swear words-across different cryptocurrencies.

In the Affect^1^ subset, our analysis encompassed various emotional dimensions, including Positive Emotion, Negative Emotion, Anxiety, Anger, Sadness, and Swear Words. In the upcoming Emotion section, we delve deeper into affective analysis. However, in this preliminary report, we provide an overview of the affective processes observed in the LIWC analysis. It can be observed in Table 3 that there is a variation in affective (good, well, new, love) content among different cryptocurrencies. Notably, Matic coin exhibits a higher level of affective language, while Ada appears to have a lower level. This distinction becomes clearer when we explore the affective subcategories including Positive tone (new, love), Negative tone (bad, wrong, too much, hate), Emotion (good, love, happy, hope), and Swear words (shit, fuckin*, fuck, damn), as depicted in Fig.  4. It becomes evident that Matic coin scores higher in Positive tone and Emotion, while Bitcoin registers a higher Negative tone. Additionally, Ripple stands out with a higher score in Swear words, indicating potential user dissatisfaction. When we further break down the Emotion category into its subsets, which encompass Anxiety (worry, fear, afraid, nervous), Anger (hate, mad, angry, frustr), and Sadness (sad, disappoint, cry), we notice that Dogecoin exhibits a higher score in Anxiety, Ripple in Anger, while most of the nine analyzed coins show similar values for Sadness. These observations contribute to our analysis and highlight the varying affective language usage across different cryptocurrencies, which we explore in greater detail in the subsequent Emotion section.

#### Want

Want words signify the author’s desires or preferences. Typically, wants are philosophically differentiated from needs by conceptualizing needs as innate and essential for survival, while wants are learned and generally linked to additional satisfaction beyond basic necessities^68^. What is important for cryptocurrency analysis in this category is the aspect of hope (want, hope, wanted, wish) as Want, or Hope, is a remarkable human ability that allows individuals to envision future events and their potential outcomes with flexibility^69^. Many users have high hopes for the future of cryptocurrency, anticipating greater benefits from their investments. From Table 3, it becomes evident that Shiba is the cryptocurrency that garners most hope among users. The range of hope scores falls between 0.19 and 0.41, with the lowest level of hope associated with Fantom. This data suggests that Shiba is particularly promising in the eyes of cryptocurrency enthusiasts, while Fantom elicits comparatively less optimism.

#### Money

Another important LIWC category is Money (business, pay, price, and market)^22^. The range of Money scores, from 2.46 for Shiba to 10.51 for Binance, indicates varying degrees of discussion or emphasis on cryptocurrency financial aspects. Notably, Binance stands out with the highest score, suggesting a significant emphasis on business and financial aspects in discussions related to this coin. Conversely, Shiba has the lowest score, indicating relatively less emphasis on these financial terms in conversations related to it. These findings offer a glimpse into the importance placed on financial and business-related aspects and potentially shed light on the perception and use of the cryptocurrencies in the broader context of market and economy.

#### Attention

At the dawn of experimental psychology, William James wrote that everyone knows what attention is. It is the taking possession by the mind, in a clear and vivid manner^70^. When users include the term Attention in their tweets, it signifies their intention to draw focus to a significant event or topic. Upon reviewing Table 3, it becomes evident that Ethereum tweets receive more attention than tweets about the other cryptocurrencies, indicating a heightened interest or emphasis on Ethereum-related matters. Conversely, tweets concerning Dogecoin appear to attract less attention when compared to tweets about the other coins, suggesting a relatively lower level of interest or engagement in discussions related to it. For Shiba, our observations indicate a prevalent sense of hope and an increased use of filler words compared to the other cryptocurrencies. This heightened expression of hope suggests a more optimistic sentiment surrounding Shiba when contrasted with the other coins. Additionally, the frequent use of filler words, including expressions like “wow”, “sooo”, and “youknow” signifies a more conversational and engaged discourse. This linguistic pattern may reflect a greater level of enthusiasm and interaction among Shiba enthusiasts.

#### Netspeak and filler

This analysis includes words commonly used in social media and text messaging, such as “bae”, “lol” and basic punctuation-based emoticons like “:)” and “;)”^65,71^. This mode of communication is widely employed by netizens during computer-mediated communication (CMC). In the context of cryptocurrency discussions, which predominantly transpire on online forums, social media platforms, and chat groups, it is customary for participants to incorporate netspeak into their interactions. Through the analysis of netspeak, researchers can understand more the degree of user engagement and interaction. Notably, the adoption of terms such as “HODL” (a deliberate misspelling of “hold”, indicating a long-term investment strategy) or “moon” (indicating an expectation of significant price increases) serves as meaningful pointers to user sentiment and active participation in discussions. In the obtained results, Matic stands out prominently with a notably high netspeak score, signaling the prevalence of internet-specific expressions and informal language related to it. The results can be found in Table 3. Fillers (wow, sooo, youknow) are non-essential sounds, words, or phrases, such as “well”, “erm” or “hmm” commonly used in speech to occupy pauses and maintain the flow of conversation without altering its meaning^65,72,73^. The filler analysis results highlight that Shiba and Dogecoin exhibit higher scores in this category compared to the other cryptocurrencies, with scores ranging between 0.02 and 0.04 for the remaining coins, as depicted in Table 3. In the sentiment analysis, it’s clear that Fantom distinguishes itself with a notably elevated positive score in comparison to the other cryptocurrencies. A consistently positive sentiment can enhance investor confidence, attract new stakeholders, and contribute to a more favorable market perception. Table 3 presents the remaining outcomes for the other cryptocurrencies.

### Sentiment and emotions analysis

Table 4 provides a detailed sentiment analysis, encompassing positive, neutral, and negative percentages for various digital coins. In the world of cryptocurrency investments, it’s common for investors to assess public sentiment before making their decisions, as highlighted in prior research^30^. Consequently, sentiment analysis has gained substantial importance on cryptocurrency markets^74^. Studies have shown that tweets expressing positive emotions wield substantial influence over cryptocurrency demand, while negative sentiments can have the opposite effect^32,33^.

Analyzing the data in Table 4, it becomes apparent that Fantom distinguishes itself by displaying a notably higher positive sentiment percentage in comparison to its digital counterparts, which strongly suggests an elevated degree of interest and enthusiasm among investors towards this digital coin.

**Table 4:** Sentiment Analysis of Cryptocurrencies (%).

	Cardano	Binance	Bitcoin	Dogecoin	Ethereum	Fantom	Matic	Shiba	XRP
Positive	53.29	54.35	46.24	34.72	55.35	60.62	58.30	41.68	50.55
Neutral	25.00	32.34	36.19	54.63	23.65	27.90	26.87	44.93	26.88
Negative	21.70	13.31	17.57	10.65	21.00	11.48	14.83	13.38	22.57

Examining opinions involves another aspect known as emotion detection. In contrast to sentiment, which can be positive, negative, or neutral, emotions offer richer categorization over personality traits by revealing experiences of joy, anger, and more. Automated methods for emotion detection have been developed to enhance the analysis of individual sentiments. The primary goal of emotion analysis is to identify the specific words or sentences conveying emotions^75^. To achieve such analysis, we employed the NRCLex library to extract and categorize emotions from text^24^. NRCLex is a Python library designed for natural language processing and sentiment analysis. The acronym stands for “Natural Resources Canada Lexicon”, and it is particularly focused on assessing sentiment in text based on word associations. NRCLex is built upon a lexicon that assigns sentiment scores to words, allowing users to analyze the sentiment of individual words, sentences, or entire documents^76^. Table 5 provides the outcomes of our emotion analysis, revealing a narrow range of results for various emotions: Anger (0.02-0.04), Surprise (0.01-0.02), Sadness (0.01-0.03), Disgust (0.01-0.02), and Joy (0.02-0.04). These consistent findings suggest that most of the coins evoke similar emotional responses, highlighting their emotional proximity.

**Table 5:** Emotion Analysis of Cryptocurrencies.

	Cardano	Binance	Bitcoin	Dogecoin	Ethereum	Fantom	Matic	Shiba	Ripple
Fear	0.0324	0.0302	0.0426	0.0232	0.0358	0.0784	0.0293	0.0250	0.0416
Anger	0.0330	0.0275	0.0362	0.0307	0.0329	0.0232	0.0301	0.0201	0.0419
Trust	0.1252	0.1205	0.0911	0.0575	0.1222	0.0833	0.1094	0.0641	0.1172
Surprise	0.0227	0.0218	0.0294	0.0199	0.0263	0.0204	0.0274	0.0193	0.0281
Sadness	0.0306	0.0250	0.0347	0.0190	0.0303	0.0215	0.0255	0.0228	0.0366
Disgust	0.0212	0.0106	0.0145	0.0112	0.0169	0.0117	0.0139	0.0121	0.0256
Joy	0.0340	0.0345	0.0322	0.0354	0.0379	0.0286	0.0444	0.0364	0.0355
Anticipation	0.2848	0.2965	0.3129	0.3752	0.2941	0.3016	0.3016	0.3467	0.2700

In contrast, when it comes to emotions such as Fear and Trust, there are more noticeable differences between the coins. For instance, when examining the sentiment of Cardano, the fear score is 0.0324, while the trust score is higher at 0.1252. Similarly, for Ripple, the fear score is 0.0416, with a trust score of 0.1172. The scores provide a difference in the emotional tones associated with these cryptocurrencies, indicating the levels of fear and trust expressed in the analyzed content.

Furthermore, the emotion of Anticipation stands out with higher scores in tweets, indicating that many users are keen on anticipating the future of these coins. Notably, Dogecoin (0.3752) and Shiba (0.3467) generate more anticipation among users when compared to the other coins.

### Readability

In this section, we pay attention to the readability of data, utilizing metrics such as the Flesch Reading Ease^25^, Flesch-Kincaid Grade Level^26^, Gunning Fog Index^27^, and Dale-Chall Readability Score^28^. Assessing readability helps distinguish between text that is straightforward to grasp and text that is complex and demands a high level of education or intelligence to comprehend. Numerous readability metrics exist for text evaluation, and we have chosen to employ the above four measures as the most widely recognized tests to assess tweets.

Table 6 presents the significant differences in readability scores across tweets related to nine different digital coins.

**Table 6:** The readability level exhibited by various cryptocurrencies.

	Flesch Reading Ease Score	Flesch-Kincaid Grade Level	Gunning Fog Index	Dale-Chall Readability Score
Cardano	58.30	10.73	12.97	12.42
Binance	56.20	10.89	12.70	12.38
Bitcoin	57.45	9.99	11.90	12.67
Dogecoin	36.99	12.00	13.03	13.39
Ethereum	56.46	11.54	13.74	12.37
Fantom	66.65	9.19	11.44	13.05
Matic	51.25	11.26	12.72	12.75
Shiba	62.18	8.57	9.83	12.98
Ripple	58.92	10.92	13.13	11.93

**Figure 5:**
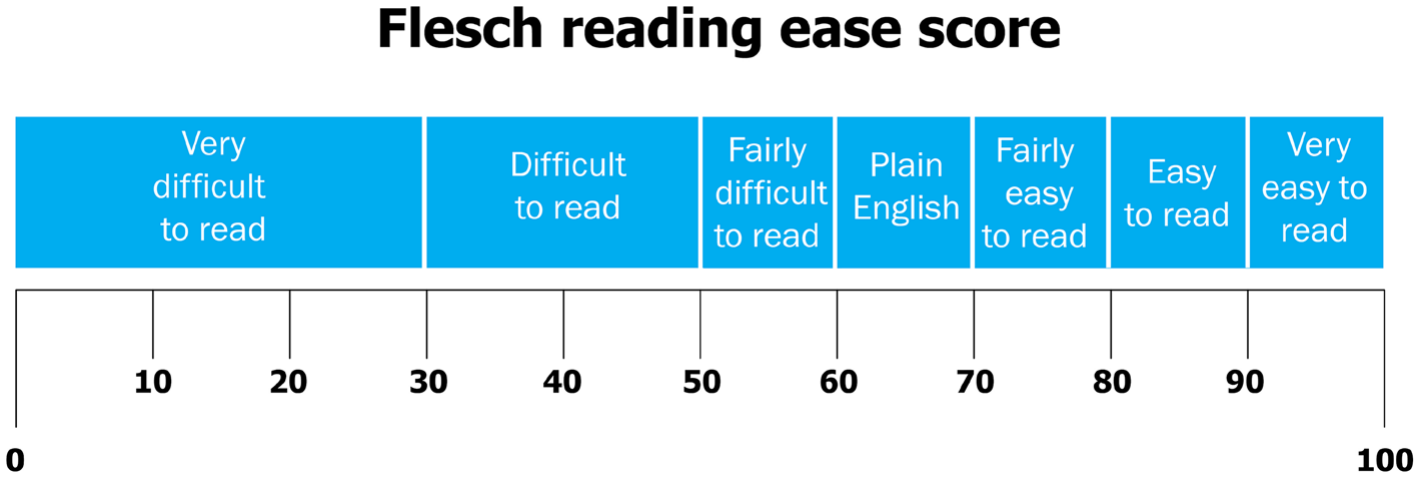
Flesch reading ease score.

**Figure 6:**
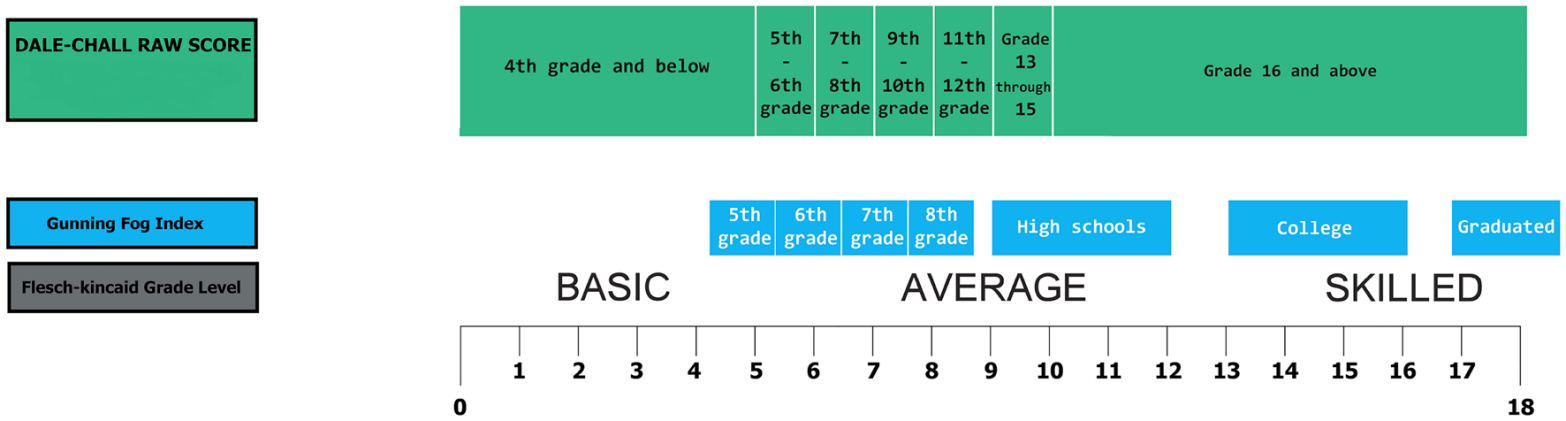
Dale-Chall Readability Score, Gunning Fog Index, Flesch-Kincaid Grade Level.

The Flesch Reading Ease score provides an indication of how easily a text can be understood, with higher scores indicating greater readability. Flesch Reading Ease score can be observed in Fig. 5. The Flesch-Kincaid Grade Level is a metric that estimates the educational grade level required to understand a piece of text based on factors like sentence length and word complexity. Analyzing the readability scores for the tweets related to each digital coin shows the linguistic complexity employed in discussions surrounding these coins. The presence of significant differences in readability scores suggests variations in the accessibility and comprehension levels required to engage with these tweets. Negative scores in some readability metrics, such as the Flesch Reading Ease and Flesch-Kincaid Grade Level, indicate higher levels of complexity, while positive scores indicate greater ease of comprehension. Refer to Fig. 6 for the necessary details to assess the readability levels of the specified analyses (Flesch-Kincaid Grade Level, Gunning Fog Index, Dale-Chall Readability Score). Table 6 provides evidence on the fact that Dogecoin possesses a notably lower score in Flesch Reading Ease compared to the other cryptocurrencies, which suggests that the communication pertaining to Dogecoin might present hurdles in accessibility and comprehension for the typical reader. Getting rid of such readability obstacles have the potential to amplify the effectiveness of communication, expand audience involvement, and cultivate heightened comprehension and acceptance of cryptocurrencies among varied stakeholders. This observation aligns with Fig. 5^77^, where we notice a pronounced level of complexity in comprehending tweets related to Dogecoin. To gain a better understanding of the varied readability levels, it’s essential to consider both Fig. 5^78,79^ and Table 6. When examining the Flesch-Kincaid Grade Level and Dale-Chall Readability in Table 6, Dogecoin emerges with higher values compared to the other cryptocurrencies, signifying an average grade level and a college reading level, respectively. Furthermore, an examination of the results pertaining to the Gunning Fog Index, as depicted in Table 6 and Fig. 6, reveals that Ethereum stands out with a higher score. This observation implies that understanding tweets related to Ethereum requires a reading comprehension level equivalent to a college education.

## Discussion

In the process of labeling our data, we identified a notable co-mention among various cryptocurrencies. We resolved this issue by excluding tweets that mentioned more than one coin or used abbreviations for coins not relevant to our research. Consequently, we focused our analysis on the specific set of cryptocurrencies pertinent to our study.

During the utilization of the LIWC software in our analysis, it became apparent that not all its components contributed substantively to the objectives of our research. In light of this, we exercised judicious discernment in the curation of our selection, focusing exclusively on those specific LIWC analyses that bore direct relevance to the concerns and interests of researchers, investors, and individuals engaged in the realm of digital marketing. Additionally, we imposed constraints on our scrutiny of emotional aspects, as certain LIWC framework components exhibited redundancy with one another.

When comparing the Analytical Thinking and Clout aspects to other LIWC features, we could see that these two scores are higher across all coins. This suggests that the tweets generally lean toward logical and formal thought. Notably, among all the coins, Fantom has the highest scores in these aspects, indicating that discussions regarding it are particularly characterized by logical and formal thinking compared to the other cryptocurrencies. After conducting a LIWC analysis, it became apparent that the highest scores were associated with two features that we considered above with concepts such as money, personal drives, and emotional affect. Furthermore, concerning the category of money and personal drives, Binance displayed notably higher scores compared to the other cryptocurrencies. Notably, Matic exhibited significantly higher levels of emotional affect when compared to the other cryptocurrencies. In contrast, other features such as Hope, Attention, Netspeak, and Filler exhibited remarkably low scores, nearly hovering around one percent, when compared to the features we previously discussed. This suggests that the tweets of users are primarily centered on analytical thinking, clout, personal drives, emotional affect, and financial matters. In the sentiment analysis, Fantom stands out with a higher positive score when compared to the other cryptocurrencies. On the contrary, Ripple registers a significantly elevated negative score. This information suggests that Fantom is generating a higher level of positive sentiment, possibly due to positive news, community sentiment, or price performance, while Ripple is experiencing more negative sentiment, which could be linked to negative news or market sentiment surrounding the coin. Emotionally, the majority of cryptocurrencies displayed a relatively modest and well-balanced emotional profile. Interestingly, there was an emphasis on the emotional category of Anticipation, and in this aspect, Dogecoin took the lead. Anticipation in this context likely refers to the expectation or excitement surrounding the future prospects, developments, or events related to these cryptocurrencies. The reason could be upcoming upgrades, partnerships, or any other factors that create a sense of anticipation among the cryptocurrency community. Regarding readability as assessed by Flesch Reading Ease, Dogecoin’s tweets scored lower on this measure. This implies that the content related to Dogecoin is relatively more difficult to read and comprehend, as its language and sentence structure are complex. Concerning Flesch-Kincaid and Dale-Chall measures, Dogecoin’s tweets received higher scores on these measures, indicating that the reading difficulty is at an average level, similar to what one might expect from a college graduate. While the Flesch-Kincaid measure estimates the U.S. grade level needed to understand the text, the Dale-Chall measure also assesses reading difficulty and is often used as a more accurate indicator for texts aimed at older audiences. Speaking of the Gunning Fog Index, Ethereum’s content, on the other hand, registered higher scores on this measure, implying a need for college-level reading proficiency. This means that content related to Ethereum is more challenging to read and understand, requiring a higher level of education and vocabulary.

## Limitations

One significant challenge encountered during the data collection phase revolved around sentences containing references to multiple cryptocurrencies. Deciphering the intended cryptocurrency from such sentences posed a considerable complexity, leading to inaccurate analysis for each coin. Therefore, these data were excluded for a more precise analysis of psycholinguistics and emotions for each coin. Additionally, the sheer volume of data presented logistical hurdles, rendering manual labeling impractical in terms of both time and financial resources.

Moreover, the dynamic nature of the cryptocurrency landscape poses another limitation, as sudden events or developments can influence user comments and sentiments, leading to shifts in behavior and sentiment. For instance, specific events like the collapse of Terra Luna or Celsius in 2022^80,81^, led to significant market price decreases. Despite efforts to mitigate these impacts through regular monitoring and updates, the inherent volatility of the cryptocurrency market presents challenges in maintaining the consistency and relevance of the dataset over time.

These limitations underscore the necessity for a cautious interpretation of the study’s findings. Future research endeavors in this domain should strive to address such methodological challenges through enhanced data collection techniques and strategies tailored to the dynamic nature of cryptocurrency discourse.

## Conclusions and future work

This paper presents a substantial dataset of English tweets related to cryptocurrencies, which were labeled using cryptocurrency keywords and abbreviations (e.g., ADA for Cardano, Ftm for Fantom, Matic for Matic, Btc for Bitcoin, Shib for Shiba, Dogecoin for Doge, Xrp for Ripple, Eth for Ethereum, and Bnb for Binance). Initially, we collected 832,559 tweets, which were reduced to 115,899 tweets after preprocessing. These tweets span from September 2021 to March 2023 and pertain to nine digital coins, namely Cardano, Bitcoin, Binance, Dogecoin, Ethereum, Fantom, Matic, Shiba, and Ripple.

This study conducted psycholinguistic and sentiment analyses on this dataset, utilizing tools such as LIWC, Emotion, Sentiment, and Readability analysis. To avoid LIWC framework redundancy, constraints were applied to the examination of emotional aspects. Our analysis revealed distinct linguistic characteristics and sentiment patterns associated with various cryptocurrencies.

Our investigation into the psycholinguistic characteristics of digital coins showed notable variations among different cryptocurrencies. Through detailed analysis of tweets related to nine distinct digital currencies, we discerned prevalent sentiments expressed by users, assessed the consistency of readability levels across various coins, and identified co-mention between different cryptocurrencies. Leveraging techniques such as psycholinguistic investigations, emotion analysis, and co-mention studies, we obtained valuable estimation of users’ perceptions and interactions.

In a broader context, our study revealed significant psycholinguistic differences among cryptocurrency data. We observed variations in sentiment and emotion analyses, as well as disparities in the readability levels associated with different cryptocurrencies. In future research, we aim to diversify our analysis techniques to delve deeper into the psychological aspects of cryptocurrency discourse. Specifically, we plan to explore sentiments of hope^69,82^ and regret^83^ in textual data using various Natural Language Processing (NLP) methodologies. Additionally, we intend to leverage Large Language Models (LLMs) to conduct psycholinguistic analyses, with an expectation to a deeper analysis of underlying linguistic patterns and emotional tones present in cryptocurrency discussions. Furthermore, our future work will involve classification algorithms with diverse machine learning approaches to distinguish bullish and bearish sentiments in comments, utilizing manual labeling for training data.

## Data Availability

The datasets generated and/or analysed during the current study are available in the GitHub repository, https://github.com/moeintash72/cryptocurrency-data-.
